# Interactions of Desmethoxyyangonin, a Secondary Metabolite from* Renealmia alpinia*, with Human Monoamine Oxidase-A and Oxidase-B

**DOI:** 10.1155/2017/4018724

**Published:** 2017-08-24

**Authors:** Narayan D. Chaurasiya, Francisco León, Yuanqing Ding, Isabel Gómez-Betancur, Dora Benjumea, Larry A. Walker, Stephen J. Cutler, Babu L. Tekwani

**Affiliations:** ^1^National Center for Natural Products Research, School of Pharmacy, The University of Mississippi, University, MS 38677, USA; ^2^Department of Biomolecular Sciences, School of Pharmacy, The University of Mississippi, University, MS 38677, USA; ^3^Programa de Ofidismo/Escorpionismo, Sede de Investigación Universitaria, Facultad de Ciencias Farmacéuticas y Alimentarias, Universidad de Antioquia, Torre 2, Laboratorio 631, Medellín, Colombia; ^4^College of Pharmacy, University of South Carolina, Columbia, SC 29208, USA

## Abstract

*Renealmia alpinia *(Zingiberaceae), a medicinal plant of tropical rainforests, is used to treat snakebites and other injuries and also as a febrifuge, analgesic, antiemetic, antiulcer, and anticonvulsant. The dichloromethane extract of* R. alpinia* leaves showed potent inhibition of human monoamine oxidases- (MAOs-) A and B. Phytochemical studies yielded six known compounds, including pinostrobin** 1**, 4′-methyl ether sakuranetin** 2**, sakuranetin** 3**, pinostrobin chalcone** 4**, yashabushidiol A** 5**, and desmethoxyyangonin** 6**. Compound** 6** displayed about 30-fold higher affinity for MAO-B than MAO-A, with Ki values of 31 and 922 nM, respectively. Kinetic analysis of inhibition and equilibrium-dialysis dissociation assay of the enzyme-inhibitor complex showed reversible binding of desmethoxyyangonin** 6** with MAO-A and MAO-B. The binding interactions of compound** 6** in the active site of the MAO-A and MAO-B isoenzymes, investigated through molecular modeling algorithms, confirmed preferential binding of desmethoxyyangonin** 6 **with MAO-B compared to MAO-A. Selective reversible inhibitors of MAO-B, like desmethoxyyangonin** 6,** may have important therapeutic significance for the treatment of neurodegenerative disorders, such as Parkinson's disease and Alzheimer's disease.

## 1. Introduction

The* Renealmia* genus (Zingiberaceae) belongs to the rare class of amphi-Atlantic plants well represented in tropical Africa and Americas [1, 2]. Several* Renealmia* species have been used for folk medicine and food on both sides of the Atlantic. Decoctions of fruits from* R. congoensis* are used to treat stomachache in Cameroon children [3]. Fruits of* R. cincinnata* are commonly used as a spice and utilized by traditional healers to treat infectious diseases in Northwest Cameroon [4]. In the Americas, several species of* Renealmia* have been reported to treat different diseases [5].* R. thyrsoidea* is used to treat skin infections associated with leishmaniasis and to reduce fever [6]. The crushed stem or infusion of* R. alpinia* is used to treat headaches, stomachaches, and body fatigue by the Amazon-Yanomami Indians [7]. Edible fruits of* R. alpinia* are valuable for taste in the Sierra Norte de Puebla (Mexico) and Ecuador [8]. In Trinidad,* R. alpinia* crushed fruits mixed with the juice of* Costus scaber* have been found effective for treatment of snakebites [9]. Otero et al. [10] reported extensive investigations on the native Northwest Colombian medicinal plants used by indigenous Embera-Katios tribes. They highlighted the use of* R. alpinia* rhizomes or/and leaves, as aqueous extracts, decoctions, or poultice for the treatment of snakebites. Moreover, the aqueous extracts of* R. alpinia* showed neutralizing effect against* Bothrops asper* venom through inhibition of proteinases present in the snake toxin [11]. Pinostrobin, the main bioactive constituent, showed inhibitory effects on the enzymatic, anticoagulant, myotoxic, and edema inducing activities of phospholipase A_2_ (PLA_2_) isolated from* Crotalus durissus* venom [12].

**Figure 1:**
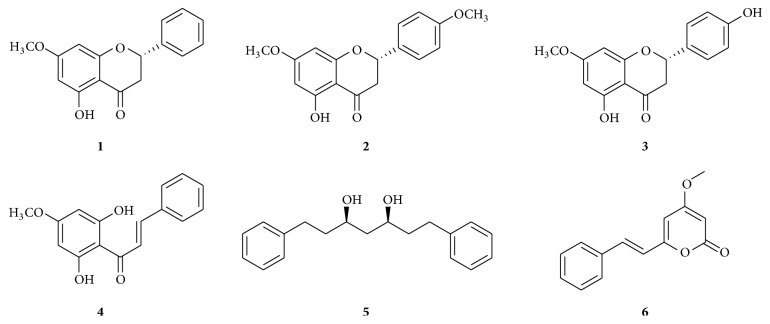
Chemical structures of* Renealmia alpinia *constituents. Pinostrobin** 1**, 4′-methyl ether sakuranetin** 2**, sakuranetin** 3**, pinostrobin chalcone** 4**, yashabushidiol A** 5**, and desmethoxyyangonin** 6.**

Additionally, the extracts of* R. alpinia* showed inhibition towards indirect hemolytic coagulant effects and proteolytic activity produced by* B. asper* venom. Pinostrobin was found to be the bioactive component in the extract responsible for this effect [13]. Recently, we explored the antinociceptive effects of methanol and aqueous extracts of* R. alpinia* in* in vivo* models. Comprehensive phytochemical analysis of* R. alpinia* yielded pinostrobin** 1**, along with two flavonoids (naringenin 7,4′-dimethyl ether** 2** and naringenin 7-methyl ether** 3**), one chalcone (2′,6′-dihydroxy-4′-methoxychalcone** 4**), one diarylheptanoid (3,5-heptanediol-1,7-diphenyl** 5**), and one kavalactone (desmethoxyyangonin** 6**) [14] (Figure 1). This was the first report on the isolation of compound** 6** from a* Renealmia* species [15].

Increasing efforts have been made to identify dual monoamine oxidases (MAOs) inhibitory and anti-inflammatory agents, which enhance cognitive functions and delay/prevent progression of neurodegenerative diseases [16–18]. Dichloromethane extract of* R. alpinia *and individual constituents isolated from this extract were evaluated* in vitro* against the recombinant human MAO-A and MAO-B. Binding interactions of desmethoxyyangonin** 6**, the most prominent MAO inhibitory constituent in* R. alpinia *extracts, in the active site of the MAO-A and MAO-B isoenzymes were investigated through enzyme-kinetics assays, enzyme-inhibitor complex binding, equilibrium-dialysis dissociation analyses, and computational molecular modeling algorithms. These studies may have implications for future research and scientifically validate traditional use of* R. alpinia *as a potential therapeutic agent for treatment of neurodegenerative disorders as well as the use of* R. alpinia *as a functional dietary benefit for the local populations.

## 2. Materials and Methods

### 2.1. Reagents and Chemicals

Pure recombinant human monoamine oxidases (MAO-A and MAO-B) enzymes overexpressed in baculovirus* (Autographa californica)* infected insect cells (BTI-TN-5B1-4) were purchased from BD Biosciences, (Bedford, MA, USA). Kynuramine bromide, 4-hydroxyquinoline, clorgyline,* R *(−) deprenyl, and DMSO were obtained from Sigma Chemicals Company (St Louis, MO, USA).* R. alpinia* extract and six evaluated compounds** 1**–**6** were obtained from the isolation procedures described previously [14]. These compounds have been kept at −20°C until evaluation. Previous to assay, to verify the stability of compounds** 1**–**6,** the spectrometric and spectroscopic analysis was done. No degradation products were detected.

### 2.2. Determination of MAOs Inhibition Activity of the Compounds

*In vitro* assays were performed to measure the inhibitory effects of* R. alpinia* dichloromethane extract and its purified compounds** 1**–**6** on human recombinant MAO-A and MAO-B activity. The dichloromethane extract (0.001 to 100 *µ*g/mL), purified compounds (10^−9^ to 10^−2^ M), and standard MAO inhibitors (phenelzine, clorgyline, and deprenyl) (10^−12^ to 10^−5^ M) were tested on human MAO-A and MAO-B enzymes [19]. Stock solutions of the test compounds/extracts were prepared in DMSO and diluted further in 0.1 M potassium phosphate buffer (pH 7.4) to obtain the desired concentrations. MAO-A and MAO-B activities were determined by fluorometric kynuramine deamination assay set up in 384-well solid white flat-bottom plates [20]. MAO-A and MAO-B inhibition activities (IC_50_ values) were determined using fixed concentration of the substrate kynuramine and varying concentrations of the test compounds or extracts. The enzyme reactions were carried out in 0.1 M potassium phosphate buffer (pH-7.4). Reaction mixture (total volume 75 *μ*L in each well) contained potassium phosphate buffer (0.1 M, pH 7.4), kynuramine (80 *μ*M for MAO-A assay and 50 *μ*M for MAO-B assay), the test compound or extract (to the desired concentration), and the enzyme (375 ng for MAO-A or 937.5 ng for MAO-B). The reaction mixtures with buffer/substrate/inhibitor were preincubated for 10 minutes at 37°C, followed by addition of MAO-A and MAO-B to initiate the reaction. The solid white microplates were incubated for 20 minutes at 37°C and the enzymatic reaction was stopped by addition of 28 *µ*L of 2 N NaOH to each well. The deaminated product of kynuramine, which spontaneously cyclizes to 4-hydroxyquinoline, was measured fluorometrically at 320 nm excitation and 380 nm emission wavelengths with a plate reader (SpectraMax M5, Molecular Devices, Sunnyvale, CA, USA). The IC_50_ values were computed by XL-Fit® from the dose-response inhibition curves.

### 2.3. Enzyme Kinetics and Mechanism of Inhibition of MAOs

The potential inhibitor desmethoxyyangonin** 6** was selected for inhibition kinetic studies with MAO-A and MAO-B. For the enzyme-kinetics analysis, assays were performed at varying concentrations of kynuramine (1.90 *μ*M to 500 *μ*M) for determination of the enzyme inhibition constants (Ki) for inhibition of MAO-A and MAO-B with compound** 6**. In addition to controls without inhibitors, two concentrations (one below and one above IC_50_ values) of the inhibitors [for MAO-A phenelzine (0.450 *μ*M and 0.900 *μ*M), desmethoxyyangonin** 6** (0.125 *μ*M and 0.250 *μ*M) and for MAO-B phenelzine (0.050 *μ*M and 0.100 *μ*M), desmethoxyyangonin** 6** (0.045 *μ*M and 0.090 *μ*M)] were tested. Results are presented as double reciprocal Lineweaver-Burk plots. The kinetic data, namely, *K*_M_, *V*_max_, and Ki values, were calculated by SigmaPlot 12.3 with enzyme-kinetics module using Michaelis-Menten equation. The results were also analyzed for the type of inhibition.

### 2.4. Equilibrium Dialysis Assay for Analysis of Binding of Desmethoxyyangonin** 6** with MAOs

Binding and inhibition of MAO-A and MAO-B with desmethoxyyangonin** 6** were further examined by incubating the enzyme with high concentrations of the inhibitor followed by extensive dialysis of the enzyme-inhibitor complex and recovery of enzyme activities. MAO-A (0.05 mg/mL protein) was incubated with desmethoxyyangonin** 6** (20 *μ*M and 100.0 *μ*M) and MAO-B (0.05 mg/mL protein) was incubated with desmethoxyyangonin** 6** (1.50 *μ*M and 20.0 *μ*M) in an enzyme incubation mixture of 1 mL containing 100 mM potassium phosphate buffer (pH 7.4). Formation of enzyme-inhibitor complex was allowed by incubation of the reaction mixtures for 20 min at 37°C. The enzyme-inhibitor mixtures after incubation were chilled, transferred to the dialysis bags, and dialyzed against 25 mM potassium phosphate buffer (pH 7.4) for 14–16 hours at 4°C (changing the dialysis buffer three times). The enzyme catalytic activities were measured before and after dialysis.

### 2.5. Time-Dependent Enzyme Inhibition Assay

MAO-A and MAO-B enzymes were preincubated for different time periods (0–15 min) with the inhibitor. Concentrations of the inhibitor tested for time-dependent inhibition with 20 *μ*g/mL MAO-A were 7.50 *μ*M (desmethoxyyangonin** 6)** and 0.600 *μ*M (phenelzine) and with 50 *μ*g/mL MAO-B were 0.40 *μ*M (desmethoxyyangonin** 6)** and 0.100 *μ*M (phenelzine). Controls without inhibitors were also run simultaneously. The enzyme activities were determined as described above.

### 2.6. Computational Methods and Software Packages

The crystal structures of MAO-A and MAO-B were downloaded from the Protein Data Bank with PDB IDs 2Z5X for MAO-A [21] and 1OJ9 for MAO-B [22]. Water molecules were removed and protein structures were preprocessed, reviewed, modified, and refined [23], using the Protein Preparation Wizard [24]. A “standard” mode of the ProtAssign algorithm was run to optimize the hydrogen-bonding (H-bond) network at neutral pH. The Impref module of Impact [25] and the OPLS_2005 force field [26–28] were employed to relax the entire structure in the recommended protein preparation protocol. The docking calculations were carried out using the Glide software [29]. The centroid of ligands harmine (HRM) in 2Z5X and 1,4-diphenyl-2-butene (1PB) in 1OJ9 was set as the center of active site. The dock ligand length was set as 25 Å and the XP module in Glide was used to rank the obtained binding poses. Prior to docking, the ligand desmethoxyyangonin** 6** was prepared using the LigPrep [30]. Molecular mechanics combined with the generalized Born surface area continuum solvation method (MM/GBSA) were employed to calculate the protein-ligand binding free energies of the docking conformations [31], using Prime [32] of the Schrodinger software suite [24]. All residues that have atoms inside 5 Å from the ligand were treated flexibly.

## 3. Results

### 3.1. MAOs Inhibitory Properties of* R. alpinia* Extract and Compounds** 1**–**6**

**Figure 2:**
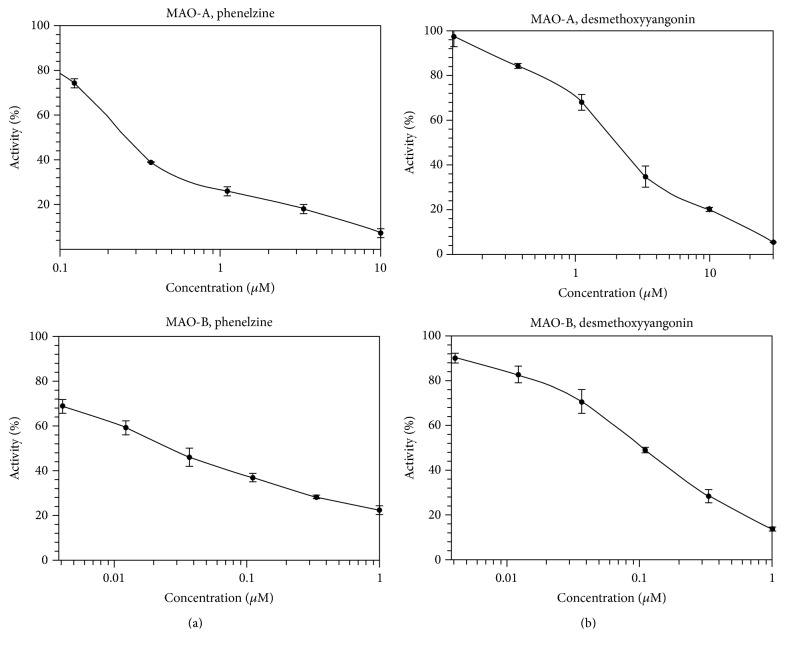
Concentration dependent analysis of inhibition of recombinant human monoamine oxidase-A and monoamine oxidase-B by desmethoxyyangonin** 6** and phenelzine.

*R. alpinia* dichloromethane extract showed significant inhibitory effect with IC_50_ values of 3.75 and 1.70 *μ*g/mL for the MAO-A and MAO-B, respectively. The extract was subjected to purification using a silica gel column chromatography, yielding six purified compounds** 1**–**6** (Figure 1). The structures of these constituents were elucidated on the basis of spectroscopic data (EIMS, ^1^H NMR and ^13^CNMR, HSQC, and HMBC), by comparison with those reported in the literature [14]. Compounds** 1**–**6** were evaluated against MAO-A and MAO-B inhibition assays. Compounds** 1**–**5** showed moderate inhibition effect; however compound** 6** exhibited potent MAO inhibition (Table 1). Noticeably, desmethoxyyangonin** 6** showed 15-fold more preferential inhibition of MAO-B than MAO-A, evidenced by the IC_50_ values of 1.850 and 0.123 *μ*M, for MAO-A and MAO-B, respectively (Table 1, Figure 2).

### 3.2. Enzyme Kinetics and Mechanism of Inhibition of MAO Isoenzymes with Desmethoxyyangonin** 6**

**Figure 3:**
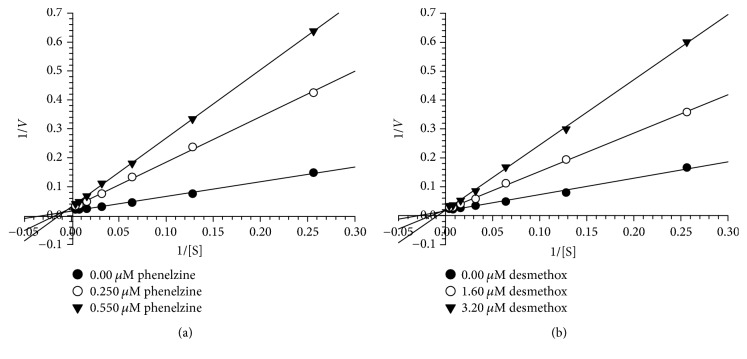
Kinetics analysis of inhibition of recombinant human MAO-A with (a) desmethoxyyangonin** 6** and (b) phenelzine; *V* = nmoles/min/mg protein and S = substrate (kynuramine) concentration (*μ*M).

**Figure 4:**
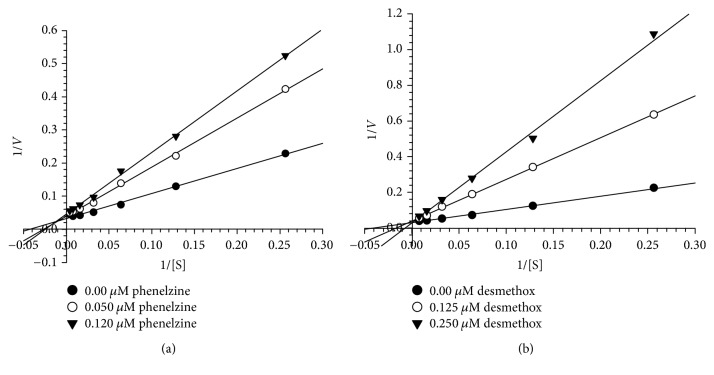
Kinetics analysis of inhibition of recombinant human MAO-B with (a) desmethoxyyangonin** 6** and (b) phenelzine; *V* = nmoles/min/mg protein and S = substrate (kynuramine) concentration (*μ*M).

We further evaluated the kinetics and mechanism of inhibition of human MAO-A and MAO-B isoenzymes by desmethoxyyangonin** 6** (Figures 3 and 4). To comprehend the type of inhibition, we examined** 6** against both MAO-A and MAO-B at varying concentrations of kynuramine, a nonselective substrate. Desmethoxyyangonin** 6** was tested at two concentrations: one above and the other below the IC_50_ value. For each experiment, three sets of assays were done at variable concentrations of the substrate: two concentrations of the inhibitor/compound and one control without inhibitors. The results are presented as double reciprocal Lineweaver-Burk plots and the kinetic data, namely, *K*_*M*_, *V*_max_, and *Ki* values, were computed by SigmaPlot 12.3 with enzyme-kinetics module using Michaelis-Menten equation (Table 2). The results suggest that desmethoxyyangonin** 6** binds as a mixed inhibitor with the human MAO-A (Figure 3). However, MAO-B inhibition by** 6** was competitive (Figure 4). The binding affinities of compound** 6** with MAO-A and MAO-B were compared with reference MAO inhibitors.

### 3.3. Analysis of Time-Dependent Enzyme Inhibition and Binding of Compound** 6** with MAOs

**Figure 5:**
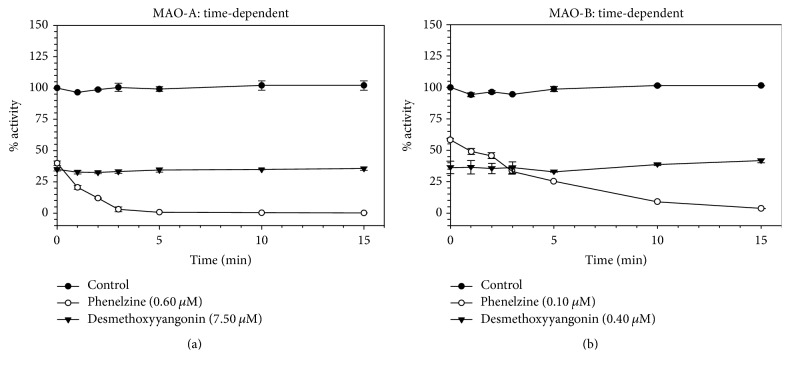
(a) Time-dependent inhibition of recombinant human MAO-A by phenelzine (0.600 *μ*M) and desmethoxyyangonin** 6** (7.50 *μ*M). Each point represents mean ± SD of triplicate values. (b) Time-dependent inhibition of recombinant human MAO-B by phenelzine (0.100 *μ*M) and desmethoxyyangonin** 6** (0.400 *μ*M). Each point represents mean ± SD of triplicate values.

**Figure 6:**
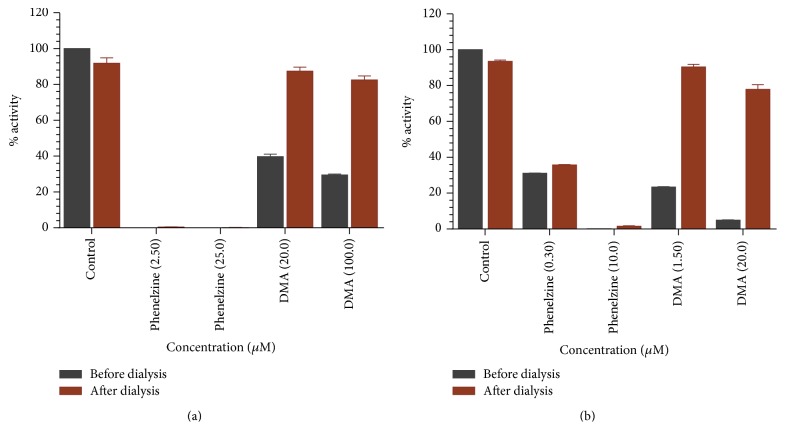
(a) Analysis of binding of phenelzine and desmethoxyyangonin** 6** with recombinant human MAO-A. Recovery of catalytic activity of the enzyme after equilibrium dialysis of the enzyme-inhibitor complex. Each bar shows mean ± SD of triplicate values. (b) Analysis of binding of phenelzine and desmethoxyyangonin** 6** with recombinant human MAO-B. Recovery of catalytic activity of the enzyme after equilibrium dialysis of the enzyme-inhibitor complex. Each bar shows mean ± SD of triplicate values. DMA = desmethoxyyangonin.

In order to examine the time-dependent binding inhibition of MAO-A and MAO-B, the enzymes were preincubated with inhibitor for the indicated time (0–15 min) at concentrations that caused nearly 60–70% inhibition (Figure 5). The control enzymes without inhibitor were also run concurrently. The results show that inhibition of MAO-A and MAO-B (Figure 5) by desmethoxyyangonin** 6** was not time-dependent. The binding characteristics of desmethoxyyangonin** 6** with MAO-A and MAO-B were examined by equilibrium dialysis to measure dissociation of the enzyme-inhibitor complex (Figure 6). MAO-A and MAO-B were incubated with high concentration of desmethoxyyangonin** 6** for 20 minutes at 37°C to allow binding of inhibitor with the enzyme and formation of enzyme-inhibitor complex. The mixtures of enzyme-inhibitor complex were dialyzed overnight at 4°C against 25 mM KHPO_4_ (pH-7.4) buffer. The enzyme activities were examined before and after dialysis. Through overnight dialysis, the recombinant human MAO-A enzyme lost about 10–15% of activity. Incubation of MAO-A with 20.0 and 100 *μ*M concentrations of desmethoxyyangonin** 6** inhibited more than 65% of the enzyme activity (Figure 6). After the dialysis, more than 90% catalytic activity of MAO-A was recovered from enzyme-desmethoxyyangonin** 6 **incubation mixtures. Similarly, MAO-B lost about 10–15% of the enzyme activity during overnight dialysis. Incubation of desmethoxyyangonin** 6 **(1.50 and 20.0 *μ*M) with MAO-B caused more than 90% inhibition of enzyme activity, which was fully recovered after dialysis (Figure 6). These observations suggest that the inhibition of MAO-A and MAO-B by desmethoxyyangonin** 6** was reversible due to dissociable nature of their enzyme-inhibitor complexes.

### 3.4. Molecular Modeling-Based Analysis of Interaction of Desmethoxyyangonin** 6** with MAO-A and MAO-B

**Figure 7:**
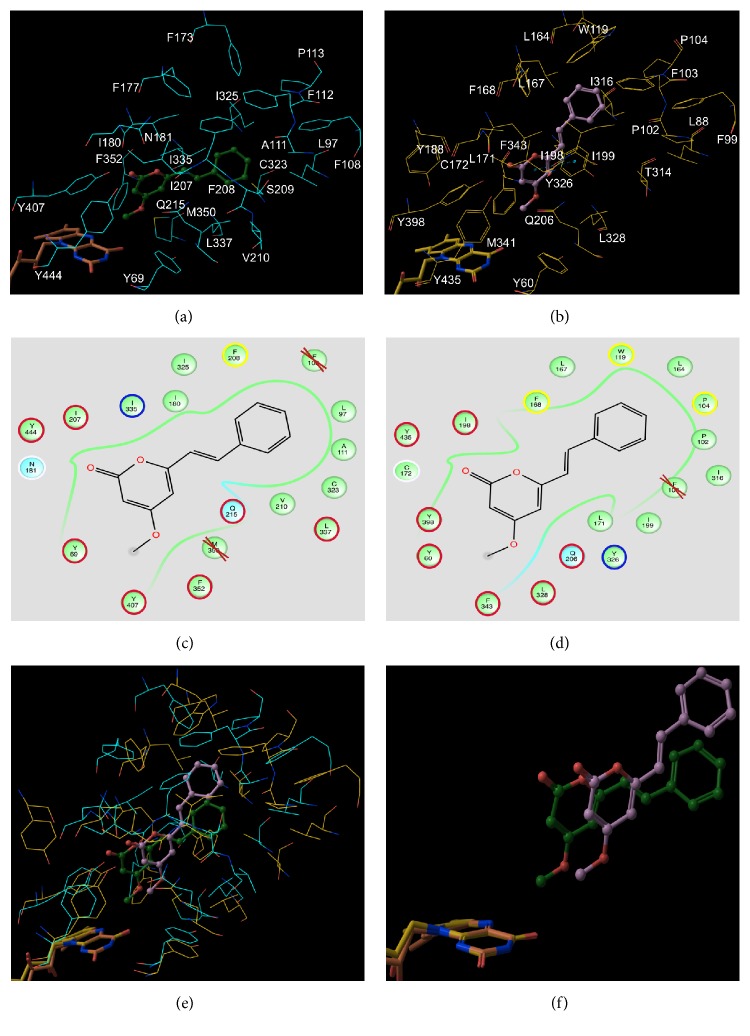
(a, c) The most preferred binding poses of desmethoxyyangonin** 6** in MAO-A and (b, d) for MAO-B. (e, f) The superposition of the complexes.

Selective inhibition of MAO-B compared to MAO-A by desmethoxyyangonin** 6** led us to the investigations on interactions of compound** 6** with the human MAO-A and MAO-B employing computational molecular modeling algorithms. The preferred binding poses of desmethoxyyangonin** 6** in MAO-A and MAO-B are shown in Figure 7, and their docking scores and MM/GBSA binding energies are listed in Table 3. The docking scores for the favored complexes with MAO-A and MAO-B are −3.89 and −7.98 kcal/mol, respectively. These scores confirm better binding of desmethoxyyangonin** 6** to MAO-B than to MAO-A and support the experimental observations on selective inhibition of MAO-B compared to MAO-A, with desmethoxyyangonin** 6**. MM/GBSA binding energies were computed as −35.10 and −62.95 Kcal/mol for the MAO-A and MAO-B complexes, respectively. These observations are consistent with their docking scores and observed experimental IC_50_ and *Ki* values. Further analysis of these interactions indicates that the pyranone moiety of compound** 6**  *π*-*π* stacks with the phenol moiety of residue Y326 in MAO-B complex (Figure 7(b)), which was correspondingly replaced by residue I335 in MAO-A complex (blue-circled in Figures 7(c) and 7(d), resp.). To facilitate this *π*-*π* stacking interaction, compound** 6** in MAO-B stays farther away from FAD than that in MAO-A (Figures 7(e) and 7(f)). The sulfhydryl hydrogen of residue C172 in MAO-B complex also interacts with pyran and ketone oxygens of** 6**, which was correspondingly replaced by residue N181 in complex MAO-A (white-circled in Figures 7(c) and 7(d), resp.). In spite of these differences, residues interacting with the pyranone moiety are very similar in both MAO-A and MAO-B complexes. Specifically, the pyranone moiety interacts with I207, Y444, Y69, Y407, F352, Q215, and L337 in MAO-A complex and correspondingly with I198, Y435, Y60, Y398, F343, Q206, and L328 in MAO-B complex, respectively. The residues, which interact with phenyl vinyl moiety of desmethoxyyangonin** 6**, are quite different in MAO-A and MAO-B complexes. Specifically, in MAO-B complex residues P104, W119, and F168 contact with phenyl vinyl moiety of desmethoxyyangonin** 6**, whereas only one aromatic residue F208 was found to be involved in interaction with phenyl vinyl moiety in MAO-A complex (yellow-circled in Figures 7(c) and 7(d)). In addition, phenyl vinyl moiety was found to have contact also with residues I180, I325, L97, A111, C323, and V210 in MAO-A complex but with residues I199, I316, L164, P102, L167, and L171 in MAO-B complex (green-circled in Figures 7(c) and 7(d)). These differences in interaction might cause rotation of phenyl vinyl moiety of approximately 180° above the (pyranone) C-C(vinyl) bond from its position in MAO-A to that in MAO-B (Figures 7(c) and 7(f)) and further imply better binding of desmethoxyyangonin** 6** to MAO-B than to MAO-A.

## 4. Discussion

Many herbs remedies contain MAO inhibitors without the unpleasant side effects. Recently, Carradori et al. [33] have listed common natural sources and the chemical features responsible for inhibition of MAO-B, justifying the potential use of folk herbs and natural products for treatment of neurodegenerative diseases. Desmethoxyyangonin** 6** is one of the main kava-pyrone derivatives known as kavalactones. Kavalactones are bioactive principles of the traditional beverage kava-kava, made from* Piper methysticum* used to treat anxiety [34]. The neurobiological activities of kavalactones primarily include modulation of gamma-aminobutyric acid type A receptors (GABA) [35]. Isolated kavalactones have shown other neurological activities potential for the treatment of neurodegenerative diseases. In fact, some potential therapeutic actions, namely, anxiety, tension, and restlessness, of standardized extracts of kava-kava roots have been attributed to kava pyrones, desmethoxyyangonin** 6,** and (+/−)-methysticin through inhibition of platelet MAO-B [36]. Similarly, kavapyrones have also been reported to show several psychotropic and neuropharmacological properties, namely, relaxation, euphoria, sleepiness, skeletal muscle relaxation, anticonvulsant properties, neuroprotection, and analgesia. Treatment of rats with kava extract caused changes in their behavior and increased the levels of dopamine in nucleus accumbens [37]. Methysticin, kavain, and yangonin have been reported to induce ERK1/2 phosphorylation, while dihydro-5,6-dehydrokavain and desmethoxyyangonin have been shown to inhibit peroxide-induced P38 phosphorylation [38]. Desmethoxyyangonin** 6** has been reported to prevent inflammation and hepatitis in mice through attenuation of lipopolysaccharide- (LPS-) stimulated inflammation in murine macrophages and LPS/D-galactosamine- (LPS/D-GalN-) induced fulminant hepatitis in mice [15]. Moreover, kavalactones such as desmethoxyyangonin** 6** have not been listed under PAINS (pan assay interference), and** 6** does not contain any PAIN-like motif [39, 40].

The presence of pinostrobin** 1** and desmethoxyyangonin** 6** in* R. alpinia* further explains the potential benefits of this plant for human health and nutrition. Anti-inflammatory properties and selective reversible inhibition of human MAO-B by desmethoxyyangonin** 6 **suggest potential therapeutic use of this kavalactone for treatment of neurodegenerative diseases like Parkinson's disease.

## 5. Conclusion

In this study, phytochemical analysis of dichloromethane extract of* R. alpinia* after fractionation and purification produced pure desmethoxyyangonin** 6**, which was further evaluated for the inhibitory effects against recombinant human MAO-A and MAO-B enzymes. Desmethoxyyangonin** 6** was identified as a prominent MAO-A and MAO-B inhibitory constituent from* R. alpinia*. The computational docking and thermodynamic analysis of MAO-A and MAO-B complexed with desmethoxyyangonin** 6** support the experimental results regarding 29-fold selective inhibition of MAO-B compared to MAO-A. The presence of prominent MAO-B constituent supports the* R. alpinia* folkloric use, and desmethoxyyangonin** 6 **can be used as a lead compound for rational design of anti-Parkinson's disease agents.

## Figures and Tables

**Table 1:** Inhibition (IC_50_ values) of recombinant human MAO-A and MAO-B by *R. alpinia* extract and isolated compounds **1**–6.^**∗**^

Samples	Monoamine oxidase-A IC_50_ (*µ*M)	Monoamine oxidase-B IC_50_ (*µ*M)	Index MAO-A/B
*Renealmia alpinia* dichloromethane extract	3.750 ± 0.283^a^	1.700 ± 0.2121^a^	2.201
Pinostrobin, **1**	23.895 ± 1.346	45.547 ± 4.314	0.524
4′-Methyl ether sakuranetin, **2**	31.400 ± 4.577	>100	—
Sakuranetin, **3**	45.482 ± 5.715	36.505 ± 6.626	1.246
Pinostrobin chalcone, **4**	6.326 ± 0.206	10.036 ± 3.237	0.610
Yashabushidiol A, **5**	>100	35.384 ± 0.121	—
Desmethoxyyangonin, **6**	1.850 ± 0.086	0.1233 ± 0.0095	15.040
Phenelzine	0.235 ± 0.0218	0.150 ± 0.0095	1.566
Clorgyline	0.0046 ± 0.003	—	—
Deprenyl	—	0.032 ± 0.012	—

^*∗*^The IC_50_ values computed from the dose response inhibition curves are Mean ± SD of triplicate observations. ^a^*μ*g/mL.

**Table 2:** Inhibition/binding affinity constants (*Ki*) for inhibition of recombinant human MAO-A and MAO-B by desmethoxyyangonin **6 **and phenelzine.^**∗**^

Compounds	Monoamine oxidase-A		Monoamine oxidase-B	
	*Ki* (nM)	Type of Inhibition	*Ki* (nM)	Type of Inhibition
Desmethoxyyangonin, **6**	922.9 ± 0.025	Mixed/reversible	31.0 ± 0.003	Competitive/reversible
Phenelzine	146.0 ± 0.009	Mixed/irreversible	110.0 ± 0.005	Mixed/irreversible

^*∗*^Values are mean ± SD of triplicate experiments.

**Table 3:** Docking scores and MM/GBSA binding energies of preferred binding poses of desmethoxyyangonin **6** in MAO-A and MAO-B.

Methods	MAO-A^a^	MAO-B^b^
DSXP^c^	−3.89	−7.98
MM/GBSA^d^	−35.10	−62.95

^a^Human monoamine oxidase-A (pdb id: 2Z5X). ^b^Human monoamine oxidase-B (pdb id: 1OJ9). ^c^Docking scores obtained by using extra precision module (Kcal/mol). ^d^Binding free energy that does not include contributions from receptor or ligand strain (Kcal/mol).
